# High-Power Distance Is Not Always Bad: Ethical Leadership Results in Feedback Seeking

**DOI:** 10.3389/fpsyg.2019.02137

**Published:** 2019-09-27

**Authors:** Zhenxing Gong, Lyn Van Swol, Zhiyuan Xu, Kui Yin, Na Zhang, Faheem Gul Gilal, Xiaowei Li

**Affiliations:** ^1^Department of Business Administration, School of Business, Liaocheng University, Liaocheng, China; ^2^Department of Communication Arts, University of Wisconsin-Madison, Madison, WI, United States; ^3^Department of Business Administration, Donlinks School of Economics and Management, University of Science and Technology Beijing, Beijing, China; ^4^Department of Business Administration, Beijing Information Science and Technology University, Beijing, China; ^5^Department of Business Administration, Sukkur IBA University, Sindh, Pakistan

**Keywords:** ethical leadership, psychological safety, nurse, power distance, feedback-seeking

## Abstract

Feedback seeking relates positively to organizational identification and task performance. However, an individual generally views seeking feedback as risky. It remains unclear whether, why, and when ethical leadership impacts on feedback-seeking behavior. This research aimed to explore the mediating role of psychological safety in the relationship between ethical leadership and nurses’ feedback seeking and to further explore the moderating effect of power distance in this mechanism. After eliminating invalid surveys, the sample included 458 pairs. The SPSS PROCESS macro was used for the data analysis. The results indicate that ethical leadership positively affected nurses’ feedback-seeking. Ethical leadership influences feedback seeking through psychological safety. With high power distance, ethical leadership significantly positively influenced psychological safety and then positively affected feedback-seeking behavior. In sum, in the context of high-power distance, ethical leadership is especially important for psychological safety and feedback-seeking behavior.

## Introduction

Constructive feedback guides or reinforces effective behaviors and reduces or stops ineffective behaviors (Steffens et al., 2018). Leaders have a general sense that feedback is good to give and receive, but many leaders are uncomfortable telling subordinates they have done well, and leaders feel even more uncomfortable telling others they have performed poorly, because some leaders think feedback should come in the form of the annual performance appraisal review (London, 2014). Thus, few subordinates would just as soon know how they did. Some subordinates may seek for the feedback in daily work. Feedback seeking can be a way to gather accurate information about oneself (Harrison and Dossinger, 2017). Research has confirmed that subordinates, who often seek feedback show strong organizational identification, higher task performance, better creative performance, and lower turnover tendency (Li and Qian, 2016). Unfortunately, more subordinates dodge evaluations of their performance and opportunities to learn how they can improve because subordinates are concerned about the potentially harmful effects of negative feedback, and they generally view feedback seeking as risky (Michiel and Frederik, 2013). As a result, even if some subordinates need external feedback, and leaders encourage feedback-seeking, subordinates often choose not to seek feedback (Stoker et al., 2012).

Given the importance of feedback-seeking, researchers have looked for ways to improve feedback-seeking behavior. As the main feedback source, the influence of leaders on employees’ feedback seeking is a hot topic (Stoker et al., 2012). In recent years, the publicity given to questionable practices in the corporate world have fostered an increasing interest in the importance of ethical issues such as ethical climates, ethical leader, etc. (Teresi et al., 2019). In particular, relying on the Social Identity Approach, people think about themselves as independent individuals, who behave on the basis of their own idiosyncratic characteristics; and in many other contexts, they are inclined to think of themselves in terms of group membership (Barattucci et al., 2017). Most importantly, when comparing ethical climates that promote prosocial behavior, such as feedback seeking, with those suggesting more individualistic behavior, it emerges that the former are more strongly associated with work performance and employees’ positive attitudes and behaviors (Pagliaro et al., 2018).

Previous studies have focused on transformational leadership, transactional leadership, and empowering leadership, among others (Anseel et al., 2015), but few studies focused on the relationship between ethical leadership and feedback-seeking. Brown et al. (2005) defined ethical leadership as the demonstration of normatively appropriate conduct through personal actions and interpersonal relationships, and the promotion of such conduct to followers through two-way communication, reinforcement, and decision-making. Several researchers have pointed out that ethical leadership describes a distinct phenomenon, both on the conceptual and empirical level (Islam et al., 2019; Zhang et al., 2019; Zhao and Xia, 2019). Ethical leadership informs subordinates of the cost of inappropriate behavior and the benefits of ethical behavior by establishing a clear reward and punishment system (Brown and Treviño, 2006).

Only a few studies have addressed the consequences of ethical leadership behavior, specifically, it remains unclear whether, why, and when ethical leadership affects feedback-seeking behavior (Ko et al., 2017). Although ethical leadership can lead to favorable outcomes, surprisingly, ethical leadership may not lead to favorite outcomes; Detert et al. (2007) found no relationship between ethical leadership and food shrinkage. Some psychological mechanisms that may explain the different effects of ethical leadership have been discussed (Brown and Treviño, 2006), but little empirical attention has been tested on psychological mechanisms (Ko et al., 2017). A clearer understanding of psychological mechanisms by which ethical leadership impacts feedback seeking is needed for the practical concerns of selecting for, developing, and motivating ethical leadership, and also be valuable for determining whether the construct developed by Brown et al. (2005) contributes something genuinely new to leadership research and practice.

With these limitations of the extant literature, this study aimed to investigate the mediating mechanism of ethical leadership on feedback-seeking behavior and to further explore the boundary conditions affecting this mechanism. We apply a social exchange model to explain the psychological mechanism between ethical leadership and feedback-seeking. The central statement in social exchange theory is that parties enter into and maintain exchange relationships with others with the expectation that doing so will be rewarding (Detert et al., 2007). The exchange of benefits, or giving something to a recipient that is more valuable to the recipient than it is to the giver, is the underlying basis for human behavior. According to the theory, each party has something of value that the other wants. The two parties decide what to exchange and in what quantities (Walumbwa and Schaubroeck, 2009). In particular, based on social exchange theory, we highlighted the link between ethical leadership and feedback seeking *via* psychological safety, especially with high-power distance for the following reasons.

First, ethical leaders use rewards and fair punishment to hold followers accountable for their ethical conduct by communicating (Zhang et al., 2019). According to social exchange theory, when subordinates receive ethical treatment and feel their leaders’ trust in them, they tend to reciprocate by feedback seeking proactively (Walumbwa and Schaubroeck, 2009). Subordinates will take risks such as seeking feedback if they trust their leader because they believe the leader will not punish them when undesirable results occur (Sağnak, 2017).

Second, since ethical leadership research is still in its infancy, studying mediating variables can help clarify impact mechanisms and deepen our understanding of ethical leadership (Brown et al., 2005). Psychological safety is seen as the belief that risky behaviors such as feedback seeking will not lead to personal harm (Frazier et al., 2017). Psychological safety reflects an individual’s belief that they will not be punished for negative consequences (Frazier et al., 2017). Ethical leaders set clear standards of conduct by communicating in daily work (Giorgi et al., 2016), not only promote subordinates ethical conduct but also behave ethically themselves (Bedi et al., 2016). The more ethical leaders exhibit ethical behavior and strictly enforce ethical standards, the more safety subordinates feel. Therefore, a positive relationship exists between ethical leadership and psychological safety (Walumbwa and Schaubroeck, 2009). As such, we draw upon social exchange theory to propose that psychological safety serves as a mediator through which ethical leadership positively influences feedback seeking.

Third, it answers the call in recent work to test the boundary conditions of the ethical leadership effect (Choi et al., 2019). Specifically, this study tested the moderation role of power distance. Power distance refers to the degree to which a society accepts unequal power distribution (Peltokorpi, 2019). Power distance affects subordinates’ sensitivity to leaders’ ethical behaviors. In a high level of power distance, ethical leaders have higher authority. On the one hand, ethical leaders need to show more ethical behaviors to consolidate their authority; on the other hand, strict behavior standards can be better implemented (Chen et al., 2013). Based on the social exchange theory, subordinates feel more obligation toward their ethical leaders for the concern and support they were shown and are thus willing to seeking feedback.

### Ethical Leadership and Feedback Seeking

Feedback seeking is a kind of initiative behavior, wherein an individual actively seeks valuable information, which can ultimately promote both individual and organizational development (Anseel et al., 2015). Cultural differences between East and West can give rise to different understandings of feedback-seeking behavior. The cultural value of “face” makes the issue of impression management more prominent in East Asia than in the West (Shin et al., 2018). It takes more effort and loss of face for subordinates to seek feedback from their leaders.

Theoretically, leadership behavior has been suggested to affect feedback seeking for two reasons. First, seeking feedback requires someone to share feedback with someone else. Therefore, leadership is naturally related to the feedback-seeking process. Second, leaders have the authority to distribute rewards and punishments, and this power is important for feedback-seeking behavior (Detert et al., 2007). Given that seeking feedback often entails personal risk, researchers generally believe that ethical leadership focuses on the need to satisfy the basic ethical requirements of a leader (e.g., fairness, honesty, integrity, and reliability). Ethical leaders need to frequently communicate with subordinates about ethical standards at work to guide and shape work behaviors accordingly (Brown et al., 2005). When ethical leaders demonstrate fairness and concern for subordinates, subordinates report higher levels of trust, and the relationship between ethical leaders and followers can be described in terms of social exchange theory (Brown and Treviño, 2006; MacDonald et al., 2013). With regard to feedback-seeking behavior, researchers have suggested that when leaders’ behaviors conform to employees’ ethical expectations, it creates an atmosphere that encourages employee participation in the enterprise and ultimately promotes feedback-seeking (Qian et al., 2017; Sağnak, 2017). In light of the above arguments, we assumed that ethical leadership would affect nurses’ feedback-seeking behavior.

Hypothesis 1: Ethical leadership would relate positively to feedback seeking.

### Ethical Leadership, Psychological Safety, and Feedback-Seeking

Ethical leadership serves as a role model (Islam et al., 2019). Ethical leaders are concerned about the job development and well-being of their subordinates in the workplace (Zhang et al., 2019). Thus, subordinates are motivated to establish positive psychological resources to improve their job performance (Sağnak, 2017). One of the positive psychological resources is psychological safety. Psychological safety means that employees feel they can freely express themselves at work without fear of criticism or retaliation (Binyamin et al., 2018). Psychological safety is characterized as a climate that generates trust and mutual respect. Subordinates feel comfortable in this climate (Cuellar et al., 2018). Ethical leaders encourage subordinates to remove obstacles preventing the expression of difficulties, and thereby creating an environment of high psychological safety. Subordinates may take risks to seek feedback if they trust their leader because they believe the leader will still encourage them when undesirable results occur (Sağnak, 2017). Therefore, a positive relationship exists between ethical leadership and psychological safety (Walumbwa and Schaubroeck, 2009).

As a result of a misunderstanding, subordinates who actively seek feedback may be viewed as trying to compete with or surpass their coworkers (Anseel et al., 2015). Therefore, when seeking feedback, subordinates sometimes worry about offending coworkers and adversely affecting interpersonal relationships (Anseel et al., 2015). A highly ethical leader will not only display various ethical qualities (e.g., honesty, self-discipline, and not being corrupt) in their communication with subordinates but also adopt various management methods to promote ethical behaviors (e.g., communication, reward, punishment, and strengthening moral standards) (Ko et al., 2017). Therefore, ethical leadership can use ethical qualities to integrate teams, create good interpersonal environments, and improve subordinates’ psychological safety. Since feedback seeking can entail interpersonal risk, psychological safety is widely regarded as an antecedent of feedback-seeking behavior (Sağnak, 2017). Given leaders’ ability to build mutually trusting interpersonal relationships and improve employees’ psychological safety, expected psychological safety can be used as a psychological mechanism to illustrate the promoting effect of ethical leadership on feedback-seeking behavior.

Hypothesis 2: Psychological safety would mediate the relationship between ethical leadership and feedback-seeking.

### The Moderating Role of Power Distance

Power distance refers to the degree to which a society accepts unequal power distribution (Choi et al., 2019). Since organizational systems and procedures cannot completely restrict the behaviors of leaders, leaders have a considerable degree of subjectivity and autonomy in their daily work (e.g., task assignment, promotion, and assessment) (Peltokorpi, 2019). Differences in power distance may lead to differences in employees’ sensitivity to ethical leadership; thus, we expect that this feature may lead to a moderation role of power distance (Chen et al., 2013).

Specifically, subordinates who work in a context of high-power distance tend to be compliant and avoid disagreements with their leader; they generally believe their leader is worthy of respect and trust (Graham et al., 2018). Since leaders with high ethical levels will more strictly abide by high ethical standards, treat subordinates fairly, and not abuse their power for personal benefit, such positive behavioral information will receive special attention from employees in organization of high-power distance and stimulate their sense of psychological safety at work (Anand et al., 2018). Subordinates who work in greater power distance organization are more likely to trust and obey leaders, and more likely to recognize the views and behaviors of leaders (Peltokorpi, 2019). Similarly, an organization with large power distance are also more likely to accept the influence of leaders’ ethical standards on the fair atmosphere and psychological safety, which has been supported from the perspective of individual and national studies (Choi et al., 2019). For example, research conducted by Kirkman et al. (2009) on the personal level shows that employees with greater power distance tend to identify and obey the behaviors of leaders more and recognize more psychological safety. Brockner et al. (2001) also found in their research at the national level that, compared with cultures with high-power distance, people in cultures with low-power distance are more inclined to pursue fair opportunities to express their opinions. In addition, the ethical leader has higher authority and more resources in an organization with high-power distance. Subordinates are more likely to recognize the leader’s authority and are more susceptible to the influence of the leader’s ethical standards and ethical behavior. Higher leader status or hierarchical position can increase the impact of ethical leadership on followers. For example, employees are more likely to pay attention to the ethical leadership of a founder CEO than of a no founder CEO. Thus, the ethical leadership of a founder CEO may be expected to have a greater effect on organizational culture than the ethical leadership of a no founder CEO (Wu et al., 2018). Thus, with high-power distance, the relationship between ethical leadership and psychological safety is strengthened.

Ethical leaders can make full use of clues and opportunities in communication with employees to create psychological safety for subordinates. In an organization with low-power distance, team members are more willing to participate in decision-making to make up for the lack of leaders, so the role of ethical leadership is weakened. For example, Kirkman et al. (2009) pointed out that teams with low-power distance are more inclined to accept the self-management organization. Where there is low-power distance, subordinates tend not to agree that power should be distributed unequally within an organization. They tend to view different positions in terms of division of labor within the enterprise and do not see managers as significantly different from themselves. Therefore, subordinates are more concerned about the possible bad consequences of a leader’s unethical behavior in low-power distance context (Tyler et al., 2000). In that context, subordinates tend to believe that they are as integral to the organization as managers are, and that they have as much responsibility as leaders. As a result, subordinates in a lower power distance setting pay less attention to the behavior of their leaders. Their sense of responsibility and psychological safety is more related to the job itself than the actions of the leader. Therefore, before deciding whether to seek feedback, subordinates will inevitably evaluate their interpersonal environment. They need a variety of cues to determine whether their feedback seeking is worthwhile and whether seeking feedback will negatively affect their image (Anseel et al., 2015). The results of their assessments will directly affect their subsequent behavioral choices. According to the characteristics of feedback-seeking behavior, we propose that power distance will significantly affect employees’ perceptions and judgments of risk and will manifest as a moderator in the effect of ethical leadership. It is proposed that:

Hypothesis 3: Power distance would moderate the relationship between ethical leadership and psychological safety, and then influence feedback seeking. With high power distance, ethical leadership significantly positively influenced psychological safety and then positively affected feedback-seeking behavior.

## Materials and Methods

### Participants and Procedures

The participants of this study were qualified bedside nurses from China, who had experience in clinical practice. Their leaders were the experienced nurses in the hospital’s nursing department or the head nurse. All participants were nurses from 10 hospitals in China. To minimize potential common method biases, separate surveys were designed for nurses and nurse leaders. The leader surveys were distributed to 83 leaders, and the subordinate surveys were distributed to 540 nurses under these leaders. Each survey was assigned a number so the leaders’ ratings could be matched with their subordinate nurses’ responses. In total, valid surveys were obtained from 72 leaders and 491 subordinates. After eliminating unmatched surveys, the final valid sample included 458 pairs (60 leaders and 458 subordinates).

Of the 458 subordinates, 89.6% were female and 10.4% were male. As for age, subordinates’average age is 26 (SD = 1.02), 70.7% (*n* = 324) were 20–30 years of age, and 95.6% (*n* = 437) were under 35.With regard to their organizational tenure, their average professional experience is 4 years (SD = 1.17), 70.4% (*n* = 322) had worked for less than 5 years and 10.8% (*n* = 49) for 6–10 years (exclusive). With regard to education, 96.9% (*n* = 444) held a bachelor’s degree or graduated from a junior college. Of the 60 nurse leaders, 86%were female, the average reported age was 39 years (SD = 3.22), and the average reported organizational tenure was 15 years (SD = 4.06).

### Ethics Statement

This study was carried out in accordance with the recommendations of the ethics committee of Liaocheng University with written informed consent from all subjects. The protocol was approved by the ethics committee of Liaocheng University (2017_7_14). All subjects have given written informed consent in accordance with the Declaration of Helsinki.

### Instruments

The questionnaire items used were developed in English. We translated all items into Chinese and then translated them back to English to ensure cross-linguistic equivalence.

#### Ethical Leadership

We used a 10-item survey developed by Brown et al. (2005) to assess ethical leadership (*α* = 0.85). An example is, “My supervisor talks about the importance of ethics.” The responses are indicated on a 7-point Likert scale ranging from 1 (strongly disagree) to 7 (strongly agree).

#### Psychological Safety

We used a 7-item survey inspired by Edmondson (1999) to assess psychological safety (*α* = 0.93); an example item is, “On my team, it is safe to take risks and take chances on new ideas.” (Edmondson, 1999). The responses are indicated on a 7-point Likert scale ranging from 1 (strongly disagree) to 7 (strongly agree).

#### Power Distance

We used a 5-item survey inspired by Robertson and Hoffman (2000) to assess power distance (*α* = 0.88). An example item is, “Managers should make most decisions without consulting subordinates.” The responses are indicated on a 7-point Likert scale ranging from 1 (strongly disagree) to 7 (strongly agree).

#### Feedback-Seeking Behavior

Feedback-seeking behavior was measured by a 5-item scale (*α* = 0.79) developed by Ashford (1986). Each leader were asked to measure how often certain types of feedback were sought by subordinates. An example is, “How often does this subordinate ask you for feedback about his/her social behaviors?” The responses are indicated on a 7-point Likert scale ranging from 1 (strongly disagree) to 7 (strongly agree).

#### Controls

This study was controlled for sociodemographic differences, including gender, education, organizational tenure, and age.

### Data Analysis

SPSS 22.0 was used for Pearson’s correlation analysis and regression analysis, and the SPSS PROCESS macro was used to calculate mediating, moderating, and conditional effects (Hayes, 2013).

## Results

SPSS 22.0 was used for Pearson’s correlation analysis and regression analysis. Procedures developed by Hayes (2013) were performed to test for mediation. Five thousand bootstrapping resamples generated 95% confidence intervals to test if there was an indirect effect of psychological safety on the relations between ethical leadership and feedback seeking. Furthermore, to test the moderating role of power distance, we used the procedure developed by Hayes (2013). Five thousand bootstrapping resamples generated 95% confidence intervals were tested to determine if the conditional moderation model was significant for psychological safety.

Table 1 shows that ethical leadership was positively related to psychological safety (*r* = 0.09, *p* < 0.05) and feedback seeking (*r* = 0.33, *p* < 0.01). Psychological safety was positively correlated with feedback seeking (*r* = 0.45, *p* < 0.01). Additionally, power distance was significantly and positively correlated with psychological safety (*r* = 0.06, *p* < 0.05).

**Table 1:** Means, standard deviations, and correlations of all measures.

	Mean	SD	1	2	3	4
1. Ethical leadership	4.79	0.76	–			
2. Psychological safety	5.62	0.81	0.09^*^	–		
3. Power distance	3.18	1.07	0.32^**^	0.06^*^	–	
4. Feedback seeking	4.82	1.19	0.33^**^	0.45^**^	−0.09	–
5. Gender	–	–	0.14^**^	−0.09	0.35^**^	0.05
6. Age	2.26	1.02	0.12^*^	0.03	0.04	0.09
7. Job tenure	2.56	1.17	0.14^**^	0.06	−0.06	0.08
8. Education	–	–	0.04	0.31^**^	0.11^*^	0.18^**^

*n = 458*.

* *p < 0.05*;

** *p < 0.01*.

As shown in Table 2, after controlling for the effect of participant demographics, ethical leadership significantly predicted psychological safety (*β* = 0.10; 95% CI: 0.01–0.20; *p* < 0.05) and feedback-seeking behavior (*β* = 0.50; 95% CI: 0.36–0.74; *p* < 0.01), as indicated by the confidence interval excluding zero. Adding psychological safety to the model, psychological safety significantly predicted feedback-seeking behavior (*β* = 0.62; 95%CI: 0.31–0.56; *p* < 0.01); ethical leadership also significantly predicted feedback-seeking behavior (*β* = 0.44; 95% CI: 0.49–0.74; *p* < 0.01). After controlling the effect of ethical leadership on feedback-seeking behavior, power distance significantly predicted psychological safety (*β* = 0.11; 95% CI: 0.06–0.21; *p* < 0.05); the interaction between power distance and ethical leadership also significantly predicted psychological safety (*β* = 0.19; 95% CI: 0.11–0.26; *p* < 0.05).

**Table 2:** Hierarchical regressions result about mediation and moderation effect.

	Feedback seeking as the dependent variable			Feedback seeking as the dependent variable			Psychological safety as the dependent variable		
	Coeff	*p*	95% CI	Coeff	*p*	95% CI	Coeff	*p*	95% CI
Ethical leadership	0.50	0.01^**^	0.36–0.74	0.44	0.01^**^	0.49–0.74	0.10	0.04^*^	0.01–0.20
Psychological safety				0.62	0.01^**^	0.31–0.56			
Power distance							0.11	0.03^*^	0.06–0.21
Ethical leadership × power distance							0.19	0.01^**^	0.11–0.26
Gender	−0.11	0.31	−0.33 to 0.11	0.09	0.37	−0.11 to 0.30	−0.30	0.01^**^	−0.45 − 0.14
Age	−0.03	0.70	−0.16 to 0.11	0.05	0.39	−0.07 to 0.18	−0.10	0.02^*^	−0.19 − 0.02
Job tenure	0.06	0.29	−0.05 to 0.18	0.01	0.86	−0.11 to 0.10	0.10	0.01^**^	0.03–0.18
Education	0.42	0.01^**^	0.20–0.63	0.03	0.77	−0.18 to 0.24	0.65	0.01^**^	0.51–0.79
*R*^2^	0.14			0.29			0.19		
*F*	14.51^**^			30.64^**^			15.27^**^		

*n = 458*.

* *p < 0.05*;

** *p < 0.01*;

*Coeff, standardized coefficients; CI, confidence interval*.

The results in Table 3 show that the total effect of ethical leadership on feedback seeking *via* psychological safety was significant (*z* = 7.24; 95% CI: 0.37–0.64; *p* < 0.05), the indirect effect was significant (Sobel *z* = 2.01; 95% CI: 0.01–0.14; *p* < 0.05). Taken together, ethical leadership affects feedback-seeking behavior *via* psychological safety.

**Table 3:** Results of the total, direct, and indirect effect of ethical leadership on feedback-seeking *via* psychological safety.

Test	Value	SE	*z*	*p*	LL 95% CI	UL 95% CI
Total effect	0.50	0.07	7.24	0.01	0.37	0.64
Direct effect	0.44	0.06	6.88	0.01	0.31	0.56
Indirect effect	0.07	0.03			0.01	0.14
Soble test	0.07	0.03	2.01	0.03		

*n = 458. LL, lower limit; CI, confidence interval; UL, upper limit*.

The results in Table 4 show that the conditional direct effect of ethical leadership on psychological safety was positive and significant in the high-power distance condition (conditional indirect effect = 0.19, SE = 0.06; 95% CI: 0.08–0.31). It was positive and not significant in the moderate-power distance condition (conditional indirect effect = 0.06, SE = 0.04; 95% CI: −0.01–0.14). Finally, it was negative and not significant under low-power distance (conditional indirect effect = 0.06, SE = 0.04; 95% CI: −0.14–0.01). The effect of moderated mediation was significant (index = 0.12, boot SE = 0.03; 95% CI: 0.06–0.18).

**Table 4:** Results for a conditional direct effect of ethical leadership on feedback-seeking across levels of power distance.

Moderator level	Mean	Effect	Boot SE	LL 95%CI	UL 95%CI
Low (M − 1 SD)	−1.07	−0.06	0.04	−0.14	0.01
Moderate level	0	0.06	0.04	−0.01	0.14
High (M + 1 SD)	1.07	0.19	0.06	0.08	0.31

*n = 458. LL, lower limit; CI, confidence interval; UL, upper limit*.

**Figure 1:**
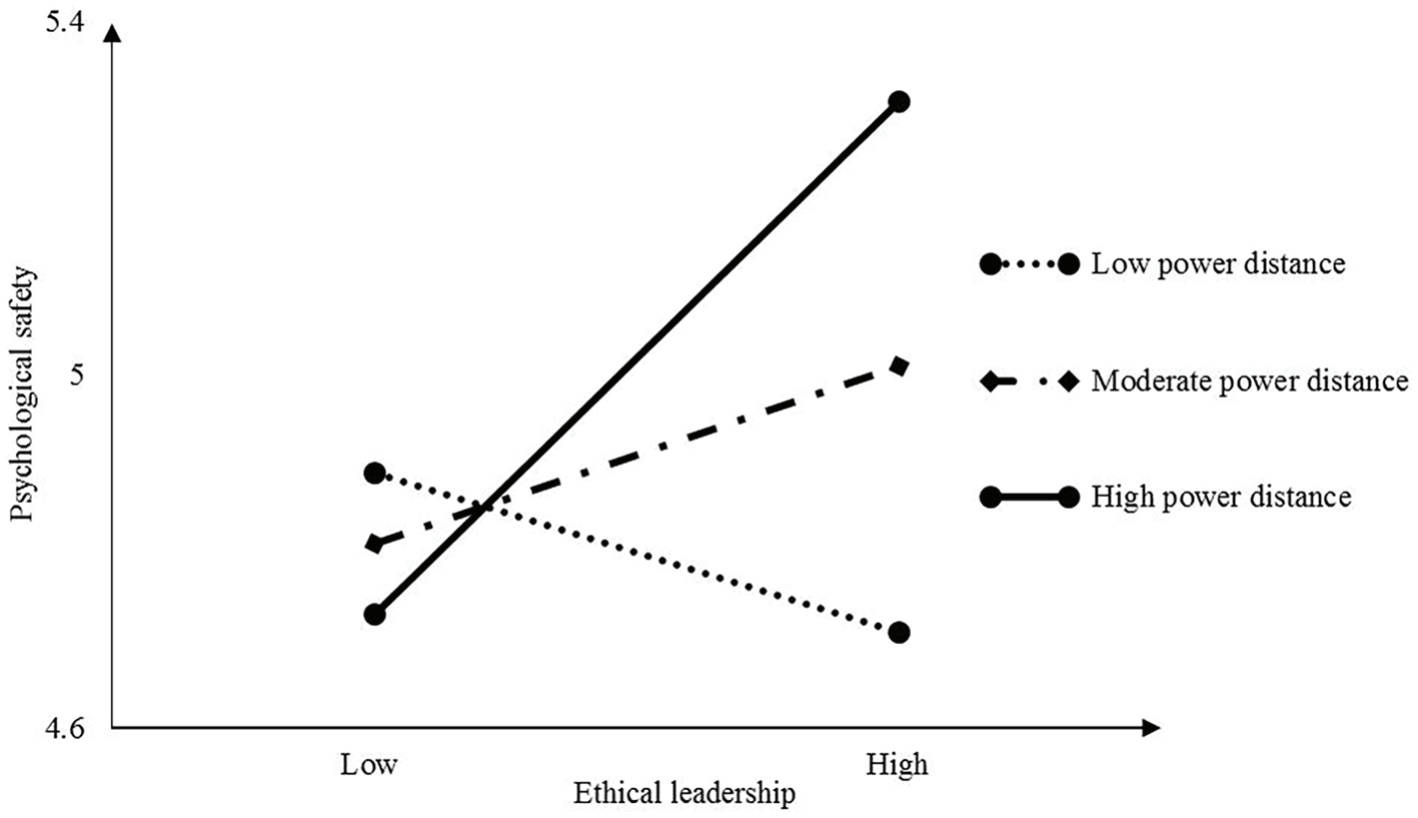
Simple slopes of ethical leadership predicting feedback seeking at low (1 SD below M) moderate, and high (1 SD above M) levels of power distance.

Figure 1 shows that the conditional direct effect of ethical leadership was positive and significant under high-power distance and was not significant under low-power distance. In summary, power distance acted as a moderator in the relationship between ethical leadership and psychological safety, and in turn, influenced feedback-seeking behavior.

## Discussion

Our results showed that psychological safety mediated the relationship between ethical leadership and nurses’ feedback-seeking behavior. Furthermore, we found that power distance served as a moderator in the relationship between ethical leadership and psychological safety. When power distance is high, ethical leadership will significantly positively influence psychological safety and then positively affect feedback-seeking behavior.

### Theoretical Contribution

The main contributions of this study are reflected in the three aspects discussed below:

First, this study successfully linked ethical leadership with feedback seeking, which fills the gap of feedback-seeking research. Prior research mainly focused on transformational leadership, authentic leadership, but few studies explore the relationship between ethical leadership and feedback seeking. This study demonstrated that ethical leadership impacts on feedback seeking positively. This finding is consistent with prior research, which found that ethical leadership was positively associated with feedback seeking from both ethical leaders and coworkers (Qian et al., 2017). De Hoogh and Den Hartog (2008) demonstrated that ethical leaders create a fair and principled structure, join them in the decision-making process, and as such share power. Seeking feedback can help to clarify when a nurse needs to learn or reorganize past information. The most important aspect of feedback-seeking behavior for nurses is that, since organizations are unable to provide timely and effective feedback, nurses must seek it out themselves (Sijbom et al., 2018). If nurses receive feedback from leaders, their positive behaviors will be more prominent and will then facilitate the adaptation process. Therefore, researchers have viewed employee feedback-seeking behavior as the first step in organizational innovation and development (Jiang and Holburn, 2018). As power owners, leaders with high ethical standards do not abuse their power but act in the interests of their subordinates as much as possible. Therefore, such leaders can have high-quality exchanges and interactions with subordinates. Such communication helps employees understand that their responsibilities are not limited in completing prescribed tasks and seeking feedback to promote enterprise development is also one of their obligations (Ahn et al., 2018).

Second, we found a new way to explore how ethical leadership influence on feedback seeking beyond traditional LMX and cost and value mechanisms (Qian et al., 2017). We found psychological safety played a meditation role between ethical leadership and feedback seeing. This finding is consistent with prior research, which found that ethical leadership was positively related to teachers’ voice behavior, and this relationship is mediated by ethical culture and psychological safety (Sağnak, 2017). Like voice, seeking feedback can also lead to interpersonal conflicts among nurses. Because of the potential risks, employees are often torn as to whether they should actively seek feedback (Chen and Hou, 2016). Based on the characteristics of feedback seeking, we regard ethical leadership as a predictive variable for stimulating feedback seeking; we constructed the relationship between the two by expanding on how ethical leadership establishes an interpersonal environment suitable for feedback seeking. As a mediating variable, psychological safety reflects the theoretical views consistently found in the literature on organizational feedback (Anseel et al., 2015). It revealed the psychological process by which ethical leaders motivate nurses to look beyond personal gains and losses, actively seek effective information, and seek to improve themselves. At the macro level, the global economic crisis began in 2008, whose effects are still ongoing (Giorgi et al., 2015). Some studies documented that problems related to the economic crisis may improve unemployment, increased workload, and affected the general health of workers by increasing the risk of health problems such as cardiovascular and respiratory diseases (Mucci et al., 2016). But, social support and job stress fully mediated the relationship between fear of the crisis and health, with all fit indices meeting their respective criteria, and with all path coefficients being significant (Mucci et al., 2016). Ethical leadership not only establishes the basic ethical norms for interpersonal communication in an organization but also strives to maintain high ethical standards in management (Giorgi et al., 2015). Therefore, as long as feedback seeking is not driven by self-interest, nurses need not worry about being alienated by supervisors and can easily express their opinions on various issues. Ethical leadership plays an important role in reducing the fear of crisis and improving psychological safety, and then increasing feedback-seeking behavior.

Third, unlike most previous studies results, these results showed that high-power distance does not always have negative effects. This result is consistent with prior research based on the China context. Prior research demonstrated that group power distance moderated the relation between leader emotional intelligence and interactional justice climate: the relation is stronger in groups with higher, rather than lower, group power distance (Rong et al., 2015). Also, power distance moderates the relationship between inclusive leadership and employee work engagement, such that the positive relationship is stronger for employees with higher power distance. In the case of high-power distance and high ethical leadership, nurses had the strongest sense of psychological safety (Carmeli et al., 2010). Nurses who work in low-power distance conditions tend to underestimate the personal risk of feedback-seeking behavior because they are more confident in their abilities. This assessment makes them more confident that their feedback-seeking behavior will get the attention of the outside world, and they largely ignore factors in the surrounding interpersonal environment (including the leader’s ethical behavior). Therefore, the incentivizing effect of ethical leadership is weakened or becomes negative. Similarly, nurses under high-power distance are likely to accept the legitimacy of leadership behavior and are affected by transformational leadership behavior. However, nurses under low-power distance do not pay much attention to the leader’s behavior, so the influence of the leader’s behavior on their perception is relatively low (Schaubroeck et al., 2007). Like prior studies’ results, power distance affects people’s sensitivity to the ethical behavior of leaders. Nurses who work in high-power distance contexts will pay more attention to the ethical behaviors of the leader, and they will be greatly encouraged by the leader’s self-discipline and by management policies emphasizing fairness, which will, in turn, influence their work attitudes and behaviors (Choi et al., 2019). The results showed that power distance significantly moderated the relationship between ethical leadership and psychological mediating variables; for nurses who perceive high-power distance, ethical leadership had a more obvious influence on psychological safety (Peltokorpi, 2019). Nurses who work in high-power distance environments believe their development is controlled externally and often feel a sense of powerfulness when confronted with power. This value orientation leads them to pay more attention to the external environment—especially, the information revealed by the behaviors of leaders, who are the main objects of feedback-seeking behavior—when assessing the risks of feedback-seeking behavior (Fock et al., 2013).

### Practical Contribution

Considering the long-term high-power distance orientation in Chinese society, the role of ethical leadership in employee feedback-seeking behavior is particularly important. Leaders should promote the development of corporate ethics. Leaders should not only practice and set ethical examples but also emphasize the establishment of moral standards and a good ethical atmosphere in the management process. Ethical leadership creates two necessary conditions for employee feedback-seeking behavior: a sense of responsibility derived from gratitude and a sense of psychological safety derived from good interpersonal relationships. Kabanoff (1991) pointed out the dilemma of leadership research: on the one hand, leadership research must emphasize the hierarchy difference between managers and ordinary employees, and on the other hand, it must study how to promote identity and cooperation between leaders and subordinates.

We believe that ethical leadership provides a certain way to solve this dilemma: that is, to give full play to the ethical appeal of leaders and use positions to make leaders become role models to inspire employees to learn and imitate, rather than just using position power as a means of management control. It is necessary for the long-term organizational innovation and development of enterprises. Leaders need to start with their own behavior, abide by the law, be disciplined, actively assume social responsibility, and reject hypocrisy. Organizations should also play a supervisory role, exposing and punishing hypocritical behaviors in a swift manner and correcting managers’ ethical misunderstandings through publicity and training. In his way, leaders will no longer pay attention to the short-term benefits of corporate hypocrisy and will recognize the long-term harm of such behaviors. Leaders need to judge the power distance to choose the appropriate management mode when they use social skills. In Chinese management context, the power distance is relatively large, so leaders should give full play to interpersonal and emotional skills to help the team build a good organizational atmosphere.

### Limitations and Future Research Suggestions

This study has some limitations. First, although the study used the method of sample pairing, individual data were still collected by self-reporting, which might not completely avoid the influence of homologous variance. At the same time, to avoid controversy, enterprises and employees will often seek to avoid being exposed to the public. Therefore, the data collected in this study might not fully reflect the true thoughts of the respondents. Future studies could attempt to collect information from multiple sources to ensure the reliability of the data. Second, this research adopted a cross-sectional study to collect data simultaneously, which can lead to a problem where the causal relationship between variables is not truly revealed. If we add a lag period to test corporate hypocrisy, the effect may be better, which would also need more follow-up verification. To improve this research, a time series test could also be adopted to make the relationships between related variables more convincing. Third, the conclusions are not referred to as a specific economic context and a specific geographic area. Future research could explore the same influencing mechanism among different countries and try to find the macro-economic reason.

## Conclusion

Our results indicate that ethical leadership influences feedback seeking *via* psychological safety. With high-power distance, ethical leadership significantly positively influenced psychological safety and then positively affected feedback-seeking behavior. This study further verified the theoretical value of ethical leadership as an independent variable; and this can provide some new ideas for future research on ethical leadership. This study also verified the positive moderating effect of power distance on the influence path of ethical leadership on psychological safety. In the context of long-term high-power distance in Chinese society, this study highlights the importance of ethical leadership for psychological security and feedback-seeking behavior.

## Data Availability Statement

The datasets generated for this study are available on request to the corresponding author.

## Ethics Statement

This study was carried out in accordance with the recommendations of the ethics committee of Liaocheng University with written informed consent from all subjects. The protocol was approved by the ethics committee of Liaocheng University (2017_7_14). All subjects have given written informed consent in accordance with the Declaration of Helsinki.

## Author Contributions

ZG, LV, and ZX provided substantial contributions to the research conception and design. ZX, NZ, and XL analyzed and interpreted the data. ZG, FG, and KY wrote the paper.

### Conflict of Interest

The authors declare that the research was conducted in the absence of any commercial or financial relationships that could be construed as a potential conflict of interest.

## References

[ref1] Ahn J. Lee S. Yun S. (2018). Leaders’ core self-evaluation, ethical leadership, and employees’ job performance: the moderating role of employees’ exchange ideology. J. Bus. Ethics 148, 457–470. 10.1007/s10551-016-3030-0

[ref2] Anand S. Vidyarthi P. Rolnicki S. (2018). Leader-member exchange and organizational citizenship behaviors: contextual effects of leader power distance and group task interdependence. Leadership Quart. 29, 489–500. 10.1016/j.leaqua.2017.11.002

[ref3] Anseel F. Beatty A. S. Shen W. Lievens F. Sackett P. R. (2015). How are we doing after 30 years? A meta-analytic review of the antecedents and outcomes of feedback-seeking behavior. J. Manag. 41, 318–348. 10.1177/0149206313484521

[ref4] Ashford S. J. (1986). Feedback-seeking in individual adaptation: a resource perspective. Acad. Manag. J. 29, 465–487. 10.5465/256219

[ref5] Barattucci M. Alfano V. Amodio S. (2017). The company judged from the inside: diversification, equity, and justice in organizations. J. Psychol. Educ. Res. 25, 65–81.

[ref6] Bedi A. Alpaslan C. M. Green S. (2016). A meta-analytic review of ethical leadership outcomes and moderators. J. Bus. Ethics 139, 517–536. 10.1007/s10551-015-2625-1

[ref7] Binyamin G. Friedman A. Carmeli A. (2018). Reciprocal care in hierarchical exchange: implications for psychological safety and innovative behaviors at work. Psychol. Aesthet. Creat. 12, 79–88. 10.1037/aca0000129

[ref8] Brockner J. Ackerman G. Greenberg J. Gelfand M. J. Francesco A. M. Chen Z. X. (2001). Culture and procedural justice: the influence of power distance on reactions to voice. J. Exp. Soc. Psychol. 37, 300–315. 10.1006/jesp.2000.1451

[ref9] Brown M. E. Treviño L. K. (2006). Ethical leadership: a review and future directions. Leadership Quart. 17, 595–616. 10.1016/j.leaqua.2006.10.004

[ref10] Brown M. E. Treviño L. K. Harrison D. A. (2005). Ethical leadership: a social learning perspective for construct development and testing. Organ. Behav. Hum. Dec. 97, 117–134. 10.1016/j.obhdp.2005.03.002

[ref11] Carmeli A. Reiter-Palmon R. Ziv E. (2010). Inclusive leadership and employee involvement in creative tasks in the workplace: the mediating role of psychological safety. Creativity Res. J. 22, 250–260. 10.1080/10400419.2010.504654

[ref12] Chen A. S. Hou Y. (2016). The effects of ethical leadership, voice behavior and climates for innovation on creativity: a moderated mediation examination. Leadership Quart. 27, 1–13. 10.1016/j.leaqua.2015.10.007

[ref13] Chen C. Liao J. Wen P. (2013). Why does formal mentoring matter? The mediating role of psychological safety and the moderating role of power distance orientation in the Chinese context. Int. J. Hum. Res. Manag. 25, 1112–1130. 10.1080/09585192.2013.816861

[ref14] Choi M. S. Cook C. M. Brunton M. A. (2019). Power distance and migrant nurses: the liminality of acculturation. Nurs. Inq. 26:e12311. 10.1111/nin.12311 31286637

[ref15] Cuellar A. Krist A. H. Nichols L. M. Kuzel A. J. (2018). Effect of practice ownership on work environment, learning culture, psychological safety, and burnout. Ann. Fam. Med. 16(Suppl. 1), S44–S51. 10.1370/afm.2198 29632225 PMC5891313

[ref16] De Hoogh A. H. B. Den Hartog D. N. (2008). Ethical and despotic leadership, relationships with leader’s social responsibility, top management team effectiveness and subordinates’ optimism: a multi-method study. Leadership Quart. 19, 297–311. 10.1016/j.leaqua.2008.03.002

[ref17] Detert J. R. Treviño L. K. Burris E. R. Andiappan M. (2007). Managerial modes of influence and counterproductivity in organizations: a longitudinal business-unit-level investigation. J. Appl. Psychol. 92, 993–1005. 10.1037/0021-9010.92.4.993 17638460

[ref18] Edmondson A. (1999). Psychological safety and learning behavior in work teams. Admin. Sci. Quart. 44, 350–383. 10.2307/2666999

[ref19] Fock H. Hui M. K. Au K. Bond M. H. (2013). Moderation effects of power distance on the relationship between types of empowerment and employee satisfaction. J. Cross-Cult. Psychol. 44, 281–298. 10.1177/0022022112443415

[ref20] Frazier M. L. Fainshmidt S. Klinger R. L. Pezeshkan A. Vracheva V. (2017). Psychological safety: a meta-analytic review and extension. Pers. Psychol. 70, 113–165. 10.1111/peps.12183

[ref21] Giorgi G. Arcangeli G. Mucci N. Cupelli V. (2015). Economic stress in the workplace: the impact of fear of the crisis on mental health. Work 51, 135–142. 10.3233/WOR-141844 24594539

[ref22] Giorgi G. Mancuso S. Fiz Perez F. Castiello D’Antonio A. Mucci N. Cupelli V. (2016). Bullying among nurses and its relationship with burnout and organizational climate. Int. J. Nurs. Pract. 22, 160–168. 10.1111/ijn.12376 25825025

[ref23] Graham K. A. Dust S. B. Ziegert J. C. (2018). Supervisor-employee power distance incompatibility, gender similarity, and relationship conflict: a test of interpersonal interaction theory. J. Appl. Psychol. 103, 334–346. 10.1016/j.leaqua.2017.11.002 29154581

[ref24] Harrison S. H. Dossinger K. (2017). Pliable guidance: a multilevel model of curiosity, feedback seeking, and feedback giving in creative work. Acad. Manag. J. 60, 2051–2072. 10.5465/amj.2015.0247

[ref25] Hayes A. F. (2013). Introduction to mediation, moderation, and conditional process analysis: a regression-based approach. J. Educ. Meas. 51, 335–337. 10.1111/jedm.12050

[ref26] Islam T. Ahmed I. Ali G. (2019). Effects of ethical leadership on bullying and voice behavior among nurses: mediating role of organizational identification, poor working condition and workload. Leadersh. Health Serv. 32, 2–17. 10.1108/LHS-02-2017-0006 30702037

[ref27] Jiang G. F. Holburn G. L. F. (2018). Organizational performance feedback effects and international expansion. J. Bus. Res. 90, 48–58. 10.1016/j.jbusres.2018.04.034

[ref28] Kabanoff B. (1991). Equity, equality, power, and conflict. Acad. Manag. Rev. 16, 416–441. 10.5465/amr.1991.4278961

[ref29] Kirkman B. L. Chen G. Farh J. L. Chen Z. X. Lowe K. B. (2009). Individual power distance orientation and follower reactions to transformational leaders: a cross-level, cross-cultural examination. Acad. Manag. J. 52, 744–764. 10.5465/AMJ.2009.43669971

[ref30] Ko C. Ma J. Bartnik R. Haney M. H. Kang M. (2017). Ethical leadership: an integrative review and future research agenda. Ethics Behav. 28, 104–132. 10.1080/10508422.2017.1318069

[ref31] Li X. Qian J. (2016). Stimulating employees’ feedback-seeking behavior: the role of participative decision making. Soc. Behav. Personal. 44, 1–8. 10.2224/sbp.2016.44.1.1

[ref32] London M. (2014). The power of feedback: Giving, seeking, and using feedback for performance improvement. New York: Routledge.

[ref33] MacDonald H. A. Sulsky L. M. Spence J. R. Brown D. J. (2013). Cultural differences in the motivation to seek performance feedback: a comparative policy-capturing study. Hum. Perform. 26, 211–235. 10.1080/08959285.2013.795572

[ref34] Michiel C. Frederik A. (2013). Understanding and encouraging feedback-seeking behavior: a literature review. Med. Educ. 47, 232–241. 10.1111/medu.12075 23398009

[ref35] Mucci N. Giorgi G. Roncaioli M. Fiz Perez J. Arcangeli G. (2016). The correlation between stress and economic crisis: a systematic review. Neuropsych. Dis. Treat. 12, 983–993. 10.2147/NDT.S98525 PMC4844458 27143898

[ref37] Pagliaro S. Lo Presti A. Barattucci M. Giannella V. A. Barreto M. (2018). On the effects of ethical climate(s) on employees’ behavior: a social identity approach. Front. Psychol. 9:960. 10.3389/fpsyg.2018.00960 29951022 PMC6008529

[ref38] Peltokorpi V. (2019). Abusive supervision and emotional exhaustion: the moderating role of power distance orientation and the mediating role of interaction avoidance. Asia Pac. J. Hum. Resour. 57, 251–275. 10.1111/1744-7941.12188

[ref39] Qian J. Wang B. Han Z. Song B. (2017). Ethical leadership, leader-member exchange and feedback seeking: a double-moderated mediation model of emotional intelligence and work-unit structure. Front. Psychol. 8:1174. 10.3389/fpsyg.2017.01174 28744251 PMC5504241

[ref40] Robertson C. J. Hoffman J. J. (2000). How different are we? An investigation of Confucian values in the United States. J. Manage. Issues 12, 34–47. 10.2307/40604292

[ref41] Rong Y. Sui Y. Yang B. (2015). The effect of leader emotional intelligence on group performance and employee attitude: mediating effect of justice climate and moderating effect of group power distance. Acta Psychol. Sin. 47, 1152–1161. 10.3724/SP.J.1041.2015.01152 (in Chinese)

[ref42] Sağnak M. (2017). Ethical leadership and teachers’ voice behavior: the mediating roles of ethical culture and psychological safety. Educ. Sci. 17, 1101–1117. 10.12738/estp.2017.4.0113

[ref43] Schaubroeck J. Lam S. S. Cha S. E. (2007). Embracing transformational leadership: team values and the impact of leader behavior on team performance. J. Appl. Psychol. 92, 1020–1030. 10.1037/0021-9010.92.4.1020 17638462

[ref44] Shin J. Kim M. Hwang H. Lee B. (2018). Effects of intrinsic motivation and informative feedback in service-learning on the development of college students’ life purpose. J. Moral Educ. 47, 159–174. 10.1080/03057240.2017.1419943

[ref45] Sijbom R. B. L. Anseel F. Crommelinck M. De Beuckelaer A. De Stobbeleir K. E. M. (2018). Why seeking feedback from diverse sources may not be sufficient for stimulating creativity: the role of performance dynamism and creative time pressure. J. Organ. Behav. 39, 355–368. 10.1002/job.2235

[ref46] Steffens N. K. Fonseca M. A. Ryan M. K. Rink F. A. Stoker J. I. Nederveen Pieterse A. (2018). How feedback about leadership potential impacts ambition, organizational commitment, and performance. Leadership Quart. 29, 637–647. 10.1016/j.leaqua.2018.06.001

[ref47] Stoker J. I. Grutterink H. Kolk N. J. (2012). Do transformational CEOs always make the difference? The role of TMT feedback seeking behavior. Leadership Quart. 23, 582–592. 10.1016/j.leaqua.2011.12.009

[ref48] Teresi M. Pietroni D. D. Barattucci M. Giannella V. A. Pagliaro S. (2019). Ethical climate(s), organizational identification, and employees’ behavior. Front. Psychol. 10:1356. 10.3389/fpsyg.2019.01356 31275196 PMC6593040

[ref49] Tyler T. R. Lind E. A. Huo Y. J. (2000). Cultural values and authority relations: the psychology of conflict resolution across cultures. Psychol. Public Policy Law 6, 1138–1163. 10.1037/1076-8971.6.4.1138

[ref50] Walumbwa F. O. Schaubroeck J. (2009). Leader personality traits and employee voice behavior: mediating roles of ethical leadership and work group psychological safety. J. Appl. Psychol. 94, 1275–1286. 10.1037/a0015848 19702370

[ref51] Wu H. Li S. Ying S. X. Chen X. (2018). Politically connected CEOs, firm performance, and CEO pay. J. Bus. Res. 91, 169–180. 10.1016/j.jbusres.2018.06.003

[ref52] Zhang N. Li M. Gong Z. Xu D. (2019). Effects of ethical leadership on nurses’ service behaviors. Nurs. Ethics 26, 1861–1872. 10.1177/0969733018787220 30078367

[ref53] Zhao H. Xia Q. (2019). Nurses’ negative affective states, moral disengagement, and knowledge hiding: the moderating role of ethical leadership. J. Nurs. Manag. 27, 357–370. 10.1111/jonm.12675 30288835

